# M(IL-4) Tissue Macrophages Support Efficient Interferon-Gamma Production in Antigen-Specific CD8^+^ T Cells with Reduced Proliferative Capacity

**DOI:** 10.3389/fimmu.2017.01629

**Published:** 2017-11-30

**Authors:** Rylend Mulder, Andra Banete, Kyle Seaver, Sameh Basta

**Affiliations:** ^1^Department of Biomedical and Molecular Sciences, Queen’s University, Kingston, ON, Canada

**Keywords:** polarized macrophages, major histocompatibility complex, interleukin-4, interferon-gamma, T cells, lymphocytic choriomeningitis virus infection

## Abstract

CD8^+^ cytotoxic T cell (CTL) responses are necessary for the lysis of virally infected cells and control of infection. CTLs are activated when their TCRs bind a major histocompatibility complex (MHC)-I/peptide complex on the surface of antigen presenting cells such as macrophages (MΦ). It is now apparent that MΦ display remarkable plasticity in response to environmental signals to polarize into classically activated M(LPS + IFN-γ) or alternatively activated M(IL-4). However, little is known about how MΦ activation status influences their antigen presentation function to CD8^+^ T cell in models of virus infection. Consequently, we tested how polarization of spleen-derived (Sp)-MΦ impacts direct presentation of viral antigens to influence effector and proliferative CD8^+^ T-cell responses. We show that M(IL-4) Sp-MΦ retain MHC-I surface expression and the ability to stimulate IFN-γ production by CTL following peptide stimulation and lymphocytic choriomeningitis virus infection to levels similar to M0 and M(LPS + IFN-γ) MΦ. However, memory CD8^+^ T cells cultured in the presence of M(IL-4) MΦ underwent significantly reduced proliferation and produced similar IFN-γ levels as coculturing with M0 or M(LPS + IFN-γ) cells. Thus, these results show a novel ability of polarized MΦ to regulate CD8^+^ T-cell proliferation and effector functions during virus infection.

## Introduction

Tissue macrophages (MΦ) comprise an important member of the mononuclear phagocyte system where they regulate inflammation, cancer, and autoimmunity (1). They are involved in innate and adaptive immune responses to invading pathogens (2), and adapt their phenotype and function in accordance with their environment through a process termed MΦ polarization (3–5). It is now understood that both tissue MΦ and bone marrow (BM)-MΦ can develop into pro-inflammatory (M1) or anti-inflammatory (M2) (6–8).

M1 or M(LPS + IFN-γ) activation occurs in response to interferon-gamma (IFN-γ) in combination with bacterial moieties, such as lipopolysaccharide (LPS) (9, 10). M(LPS + IFN-γ) MΦ exhibit elevated secretion levels of nitric oxide (NO), and pro-inflammatory cytokines including tumor necrosis factor (TNF)-α, and IL-1β (11). Phenotypically, M(LPS + IFN-γ) cells express major histocompatibility complex (MHC)-II, and the costimulatory molecules CD80 and CD86 (6) to stimulate CD4^+^ T-cell proliferation (6, 12). M(LPS + IFN-γ) cells have been studied for their anti-bacterial, anti-viral immunity (13–17).

On the other hand, M2 cells are subdivided into M2a, M2b, M2c, and M2d depending on their environmental stimulus. The most studied subclass, M2a, is induced with interleukin IL-4 or IL-13 (9, 10). M2a or M(IL-4) MΦ upregulate Arginase-1 expression (11), and express high levels of mannose receptor (CD206) and chitinase-3-like protein 3 (Chi3l3) (6, 18). As such, M(IL-4) MΦ are widely considered regulatory and reparative cells (19). However, unchecked expansion of M2 MΦ can cause severe pathologies (19). For example, during chronic hepatitis C virus (HCV) infection, circulating and liver monocytes convert to an M2-like state resulting in fibrosis development (20). It is therefore important to study MΦ polarization during virus infection as a strategy to unlock MΦ targeting therapeutics to limit virus-associated damage (17).

During lymphocytic choriomeningitis virus infection (LCMV), MΦ support viral replication, process, and present viral antigens to activate CD8^+^ T cells (21–26). Activated CD8^+^ T cells proliferate, gradually acquire cytotoxic T lymphocyte (CTL) effector function and home to the site of infection to secrete IFN-γ and lyse virally infected upon recognition of viral epitopes on MHC-I (27). It is known that M(IL-4) peritoneal MΦ and BM-MΦ inhibit OT-II proliferation in a signal transducer and activator of transcription (STAT)-6-dependent fashion (28). Moreover, in a murine norovirus infection model, helminth-induced M2 cells inhibit CD8^+^ T-cell proliferation (29). Yet, how polarized MΦ engage CD8^+^ T cells to control proliferation and functions during RNA virus infection remains unexplored. Here, we report on a novel finding supporting a dichotomized regulatory role of M(IL-4) tissue MΦ where they can inhibit CD8^+^ T-cell proliferation without affecting their IFN-γ production after peptide-specific antigen presentation.

## Results

### Phenotypic and Functional Characterization of Activated Spleen-Derived (Sp)-MΦ

**Figure 1:**
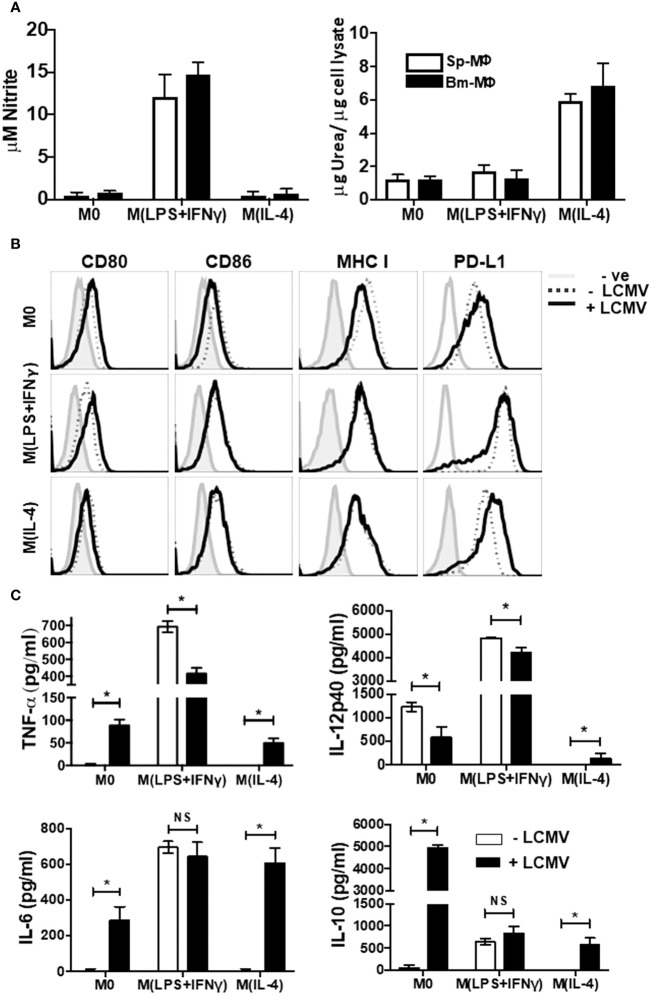
Immunophenotyping of Polarized Macrophages. Activated BM-MΦ or Sp-MΦ populations were polarized into M(LPS + IFN-γ) (25 ng/ml IFN-γ + 100 ng/ml LPS), or M(IL-4) (20 ng/ml IL4) or left un-stimulated. **(A)** Nitrite detection after BM-MΦ or Sp-MΦ were polarized into M(LPS + IFN-γ) or M(IL-4) or left un-stimulated (left panel). Supernatants were collected before testing them for nitrite production using the Greiss reaction. The OD was measured using Varioskan plate reader to quantify nitrite production after comparing the values to the standard curve. In the right panel, urea production was measured in polarized BMinfected with LCMV-WE (MOI 5M-MΦ and Sp-MΦ samples to monitor arginase activity indicative of M(IL-4) polarization. Values are represented as μg urea corrected to μg cell lysate. Data shown and error bars are the mean ± SD from one representative experiment out of three. **(B)** Staining profiles of activated polarized BM-MΦ and Sp-MΦ populations that were either controls or infected with LCMV-WE (MOI 5 for 24 h). Histograms show surface staining for CD80, CD86, MHC I or PD-L1 in the various MΦ populations compared to the isotype control (-ve). Data shown are representative from one of two experiments. **(C)** Cell supernatants from LCMV uninfected or LCMV infected (24 h) polarized Sp-MΦ were subjected to ELISA for quantification of TNF-α, IL-12p40, IL-6 and IL-10. Graphical data show mean ± SD from two independent experiments containing two experimental replicates.

Nitrite and urea production are known to be effective functional measures of M(LPS + IFN-γ) and M(IL-4) polarization, respectively (6, 8). Therefore, to demonstrate plasticity of Sp-MΦ, we characterized the biochemical properties profiles of polarized BM-MΦ and Sp-MΦ following IFN-γ (16 h) + LPS (8 h) or IL-4 stimulation (24 h). In agreement with previous publications, BM-MΦ and Sp-MΦ induce significant nitrite production after M(LPS + IFN-γ) stimulating conditions (Figure 1A: left panel), while producing urea following IL-4 treatment (Figure 1A: right panel) confirming previous published data (6, 8). Thus, both BM-MΦ and tissue-derived Sp-MΦ show similar biochemical profiles when polarized into M(LPS + IFN-γ) and M(IL-4) status as reported previously (8).

Professional antigen presenting cells (pAPC) such as MΦ are needed for the activation of adaptive immune cells (30), as they are involved in antigen presentation *via* both MHC-I and MHC-II as well as their expression of costimulatory molecules (31). Nevertheless, LCMV has evolved mechanisms to interrupt APC activation and costimulatory molecule expression (32). Therefore, in order to assess the ability of polarized Sp-MΦ to engage CD8^+^ T-cell receptors, we characterized surface expression of activated Sp-MΦ markers following 24 h of LCMV infection (Figure 1B). With regard to CD80 expression, M0 and M(LPS + IFN-γ) cells increased surface levels following viral infection, while M(IL-4) cells expression of CD80 remained largely unchanged (column 1). Interestingly, M0 cells slightly decreased CD86 expression following LCMV infection compared with M(LPS + IFN-γ) and M(IL-4) cells where no change was detected (column 2). M0 cells exhibited slight MHC-I reduction but not M(LPS + IFN-γ) or M(IL-4) Sp-MΦ (column 3). In addition, we also assessed expression of the inhibitory molecule PD-L1 (column 4). We observed that M(LPS + IFN-γ) cells expressed the greatest levels of PD-L1, while M0 and M(IL-4) had similar expression levels, which confirmed data in BM-MΦ published by another group (33). LCMV infection increased expression of PD-L1 in M0 and M(IL-4), while reduced expression in M(LPS + IFN-γ) Sp-MΦ. These data demonstrate that polarized cells are not negatively affected by LCMV infection when considering CD80/86 or MHC-I expression, while LCMV increases inhibitory molecule PD-L1 expression in M2 and M0 cells, but not M(LPS + IFN-γ).

To characterize further the functional profile of polarized cells, we investigated the release of pro- and anti-inflammatory cytokines in uninfected and LCMV-infected (24 h) Sp-MΦ. As expected, for the secretion of the cytokines TNF-α and IL-6 (Figure 1C), M0 and M(IL-4) cells were poor, while M(LPS + IFN-γ) stimulation produced substantial levels agreeing with what has been described previously (34). Interestingly, 24 h post-LCMV infection, M0 and M(IL-4) cells both significantly increased production of TNF-α and IL-6. Moreover, M(LPS + IFN-γ) cells had reduced production of TNF-α after infection but were still producing significantly higher amounts than M0 and M(IL-4). No changes in IL-6 secretion were observed with M(LPS + IFN-γ) after the infection.

Lymphocytic choriomeningitis virus infection significantly decreased production of IL-12p40, in M0 and M(LPS + IFN-γ) cells while the opposite is true for M(IL-4), where production levels increased. Collectively, these data point to LCMV-promoting M(IL-4) cells to acquire a mixed M(LPS + IFN-γ)/M(IL-4) phenotype considering the ability to produce pro-inflammatory cytokines post-infection. For the anti-inflammatory cytokine IL-10, LCMV infection increased secretion in all subsets; however, M(LPS + IFN-γ) and M(IL-4) produced substantially less amounts than M0 infected cells (Figure 1C).

### M(IL-4) Sp-MΦ Present SIINFEKL Peptide Bound to MHC-I at Lower Levels Compared with M(LPS + IFN-γ)

**Figure 2:**
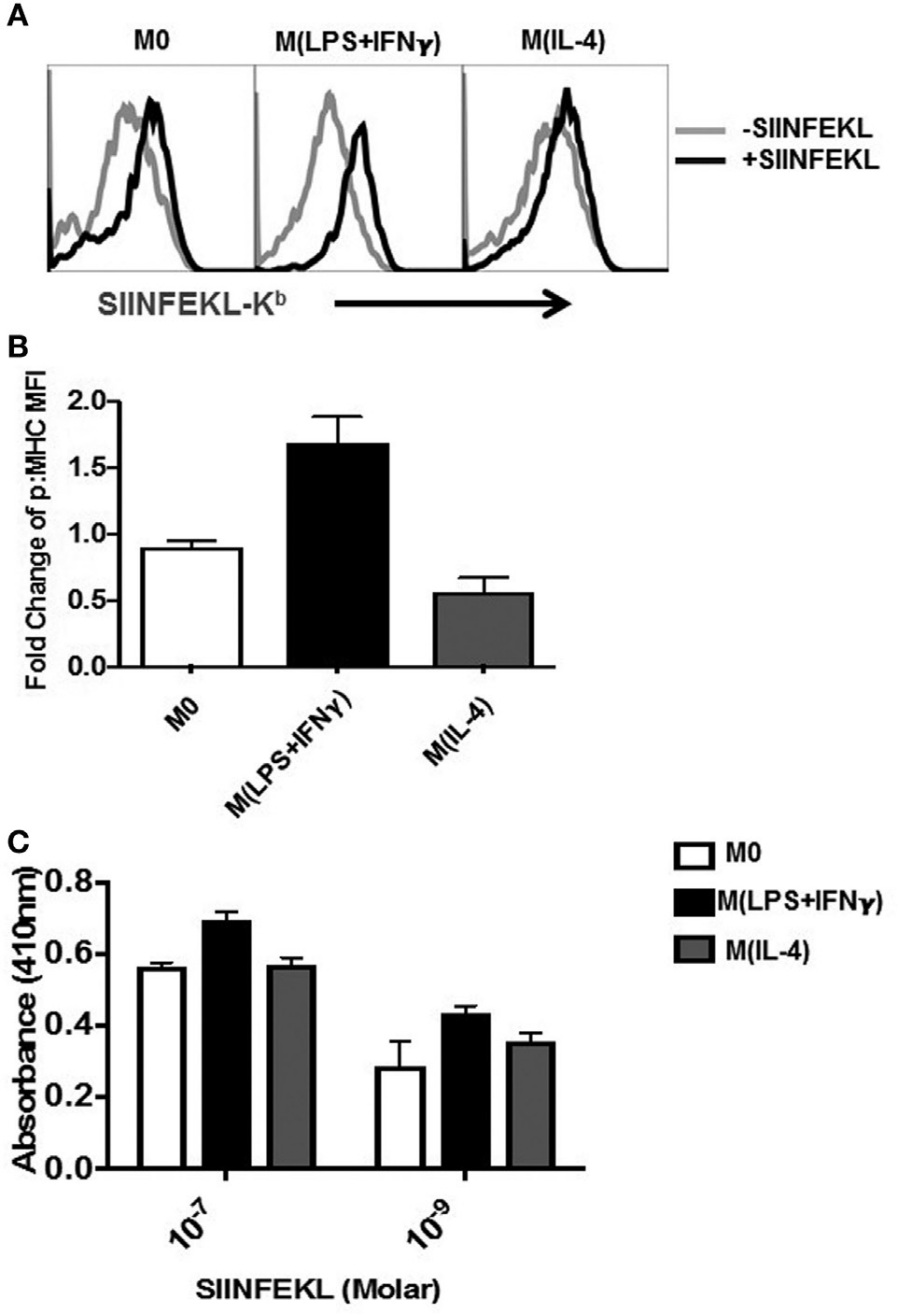
Detection of SIINFEKL peptide bound to MHC-I on MΦ. Sp-MΦ were polarized into either M(LPS + IFN-γ), M(IL-4) or left untreated (M0) and pulsed with SIINFEKL (10^−7^M) for 2 h at 37°C. **(A)** Cells were stained with 25-D1.16 monoclonal antibody, which detects SIINFEKL bound to H2-K^b^ MHC-I (p:MHC) before acquisition using FCM. The data are demonstrative histograms from one of three representative experiments. **(B)** Fold change in MFI of detected ab staining was calculated by comparing 25D staining in SIINFEKL pulsed versus unpulsed controls. Graphical data show mean ± SD from three independent experiments. **(C)** Cells were pulsed 10^−7^ or 10^−9^ M SIINFEKL for 2 h at 37°C before coincubation with the T-cell B3Z hybridoma for 18 h (1:1 ratio). The detection assay was carried out as described in Section “Materials and Methods” and OD was measured at 415 nm. Graphs depicting mean ± SD from three experimental replicates. MFI, mean fluorescent intensity; MHC, major histocompatibility complex.

Having observed substantial levels of MHC-I expression on all MΦ, we questioned to what extent polarized MΦ can bind and present MHC-I peptides. For this, we utilized the 25-D1.16 monoclonal antibody, which recognizes the SIINFEKL peptide only when bound to H2-K^b^ MHC-I (p:MHC) (35). Representative staining of unpulsed and SIINFEKL-pulsed all MΦ (1 h) histograms depicted in Figure 2A demonstrate that each population of Sp-MΦ are able to display p:MHC on their surface. Measuring the fold change in mean fluorescent intensity (MFI) over unpulsed controls revealed M(LPS + IFN-γ) were best at binding and presenting the peptide and that Sp-M(IL-4) cells were the least efficient (Figure 2B). This suggests that the polarized all MΦ subsets should be able to present H2-K^b^ restricted epitopes to CD8^+^ T cells to varying degrees.

Based on the above observations (Figures 2A,B), we reasoned that this would translate to differential abilities to activate CD8^+^ T cells by the MΦ population. To test this, we employed the CD8^+^ T-cell hybridoma system for which inducible *Lac-Z* is under the NFAT enhancer that binds the IL-2 promoter (36, 37). In this system, SIINFEKL-K^b^-specific TCR ligation results in binding to the IL-2 promotor and expression of downstream *Lac-Z* that can be detected by colorimetric changes (38). Using two different concentrations of the SIINFEKL peptide (10^−7^ and 10^−9^ M), we observed that M(LPS + IFN-γ) MΦ populations elicited B3Z activation better than M0 and M(IL-4) (Figure 2C). Yet, M(IL-4) were still very proficient in activating B3Z T cells indicating that M(IL-4) cells retain sufficient antigen presentation capabilities that was close to M0 cells.

### M(IL-4) MΦ Effectively Stimulate Epitope-Specific CD8^+^ T Cells to Synthesize IFN-γ

**Figure 3:**
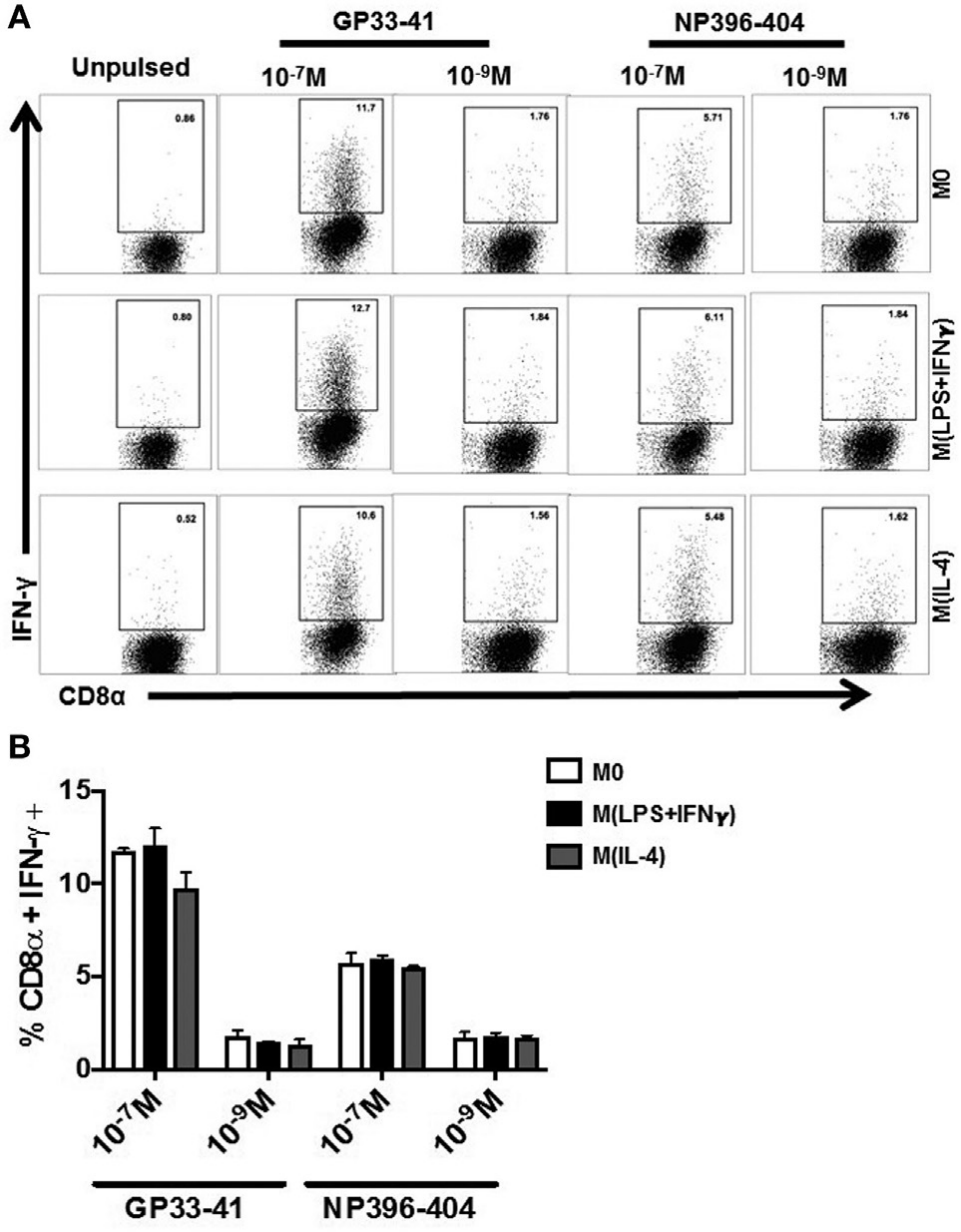
Stimulation of epitope-specific CD8^+^ T cells by polarized MΦ *via* antigen presentation. Sp-MΦ were polarized into either M(LPS + IFN-γ), M(IL-4) or left untreated (M0) and were pulsed with GP33-41 or NP396-404 (10^−7^ or 10^−9^ M) for 1 h in serum free medium. Cells 1 × 10^5^ were washed and cocultured with splenocytes from LCMV-infected mouse (20,000 pfu; day 8 p.i). Sp-MΦ were cocultured at a ratio of 10 (splenocytes): 1 Sp-MΦ for 6 h at 37° followed before CD8α and IFN-γ on CD3^+^ gated cells. As a negative control, unpulsed polarized Sp-MΦ were cocultured with splenocytes as described above. The percentage of CD8α^+^ IFN-γ^+^ from the dot plots in **(A)** were grouped in **(B)** where the data shown are the mean ± SD from three experimental replicates. LCMV, lymphocytic choriomeningitis virus infection.

Recently, it was shown that splenic marginal zone MΦ are responsible for activating an LCMV-specific CD8^+^ T cells when left unprimed by DC (39). Therefore, we extended our testing into the LCMV system, which includes H2-K^b^ and H2-D^b^ binding epitopes in H-2^b^ mice (40). Polarized Sp-MΦ were pulsed with either GP33-41 or NP396-404 then cocultured with splenocytes isolated from LCMV-infected mice (2 × 10^5^ pfu i.p.), 8 days post-infection. The ability of virus-specific CD8^+^ T cells to produce IFN-γ after *in vitro* re-stimulation with the ICS assay was used to assess the polarized Sp-MΦ antigen presentation functions (Figure 3).

We found that the percentage of IFN-γ antigen-specific CD8^+^ T cells remained constant irrespective of MΦ activation status (Figures 3A,B). Moreover, in agreement with immunodominance hierarchy reported for this model (41), there was a greater percentage of IFN-γ-secreting GP33-41-specific CD8^+^ T cells than NP396-404 T cells (Figures 3A,B). This finding was identical irrespective of the activation MΦ state even at low-peptide concentrations of 10^−9^ M. We further tested IFN-γ induction in LCMV-memory-specific CD8^+^ T cells using the LCMV GP33-41 peptide. After *ex vivo* stimulation by GP33-41 pulsed MΦ, we found that M(IL-4) cells were able to elicit similar activation of the memory T cells compared with M0 (data not shown). Therefore, we were able to demonstrate that M(IL-4)-MΦ polarization does not negatively influence their ability to stimulate activation and release of IFN-γ from antigen-specific effector or memory CD8^+^ T cells after virus infection.

### Evaluating the Ability of Activated MΦ to Present Viral Antigens after Infection

**Figure 4:**
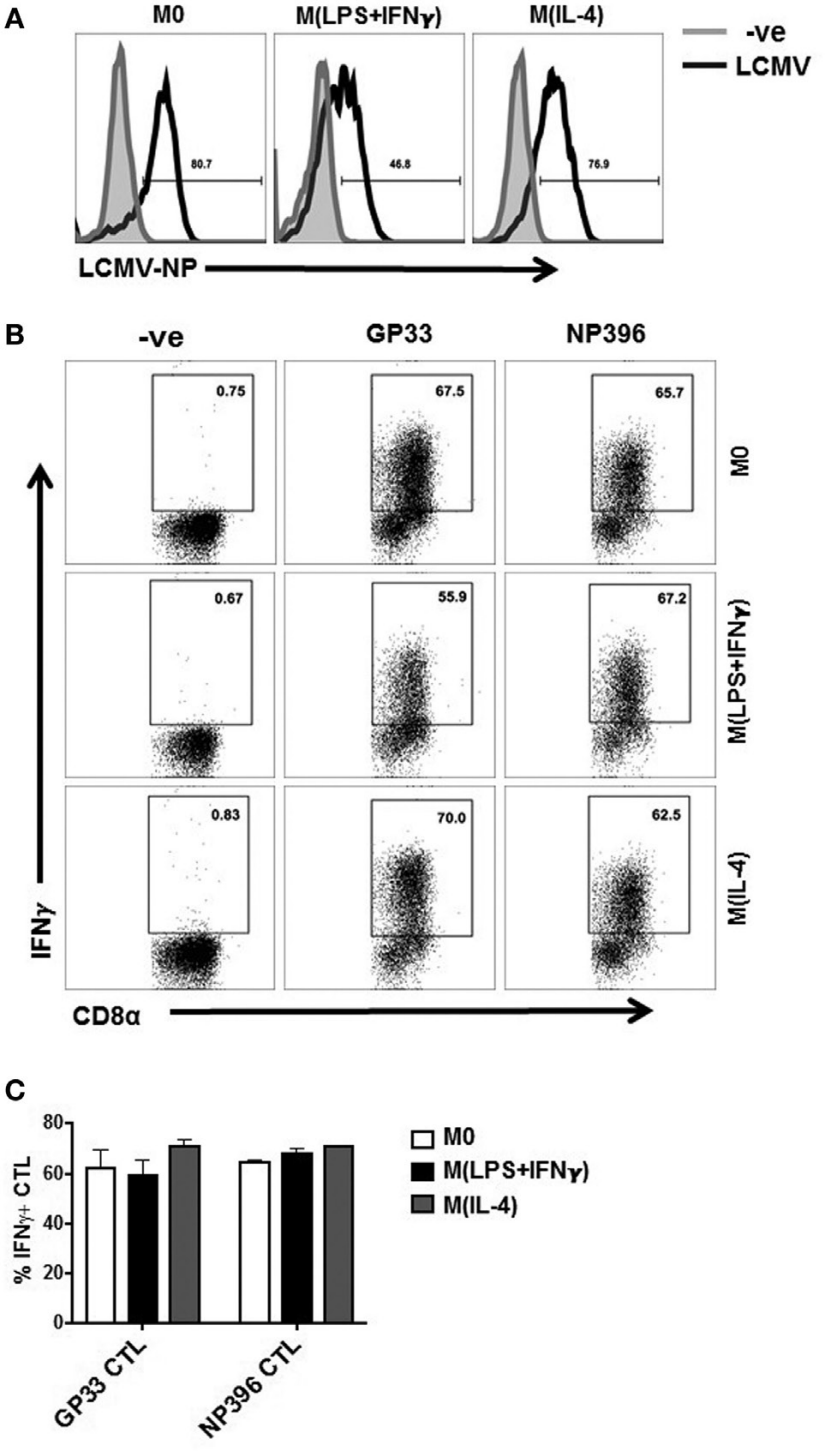
Presentation of viral epitopes to CD8 T cells by LCMV-infected MΦ. **(A)** Polarized Sp-MΦ were infected with LCMV-WE (MOI 5) before testing for expression of LCMV-NP 24 h later. **(B)** In parallel, the same infected Sp-MΦ or uninfected negative controls were tested for their ability to directly present the LCMV GP33-41 or NP396-404 epitopes to their specific CTL cultures by assaying for IFN-γ production after *in vitro* stimulation by ICS. **(C)** Graphical representation of dot plots from B showing mean ± SD from three repeats. CTL, cytotoxic T lymphocyte; LCMV, lymphocytic choriomeningitis virus infection.

To assess whether the functional abilities with regard to antigen presentation were retained by the MΦ during viral infection, we infected Sp-MΦ with LCMV (MOI 5), and assessed their ability to present LCMV antigens to epitope-specific CTL. It was clear from the data (Figure 4A) that LCMV was to infect the polarized MΦ *in vitro* and initiate its replication cycle as evident by LCMV-NP detection 24 h post-infection (Figure 4A). This protein was not detected immediately during the first hour of infection and needed to accumulate for approximately 8 h post-infection to be detected at significant levels due to the increased number of copies as a result of viral replication (data not shown), confirming our previous published data (42). Notably, we detected a reduction in LCMV-NP expression in M(LPS + IFN-γ) polarized cells compared with M0 and M2 (Figure 4A) indicating that M(LPS + IFN-γ) cells were likely inhibiting viral replication as described elsewhere.

After 24 h of LCMV infection, we assessed the antigen presenting capacity of polarized MΦ compared with M0 to epitope-specific CTLs. From the data shown in (Figures 4B,C), M(LPS + IFN-γ) cells activated GP33-41- and NP396-404-specific CTLs to levels similar to M0 and M(IL-4) cells. Thus, to our surprise M(IL-4) cells were very potent stimulators IFN-γ release from both GP33-41- and NP396-404-specific CD8^+^ T cells following viral infection. Thus, despite the prevailing dogma of M(IL-4) MΦ perceived to be poor antigen presenting cells when interacting with CD8^+^ T cells, we discovered that they were proficient in presenting either peptides or processing viral antigens in different model systems.

### M(IL-4) Sp-MΦ Poorly Support Epitope-Specific CD8^+^ T-Cell Proliferation When IL-2 Levels are Limiting

**Figure 5:**
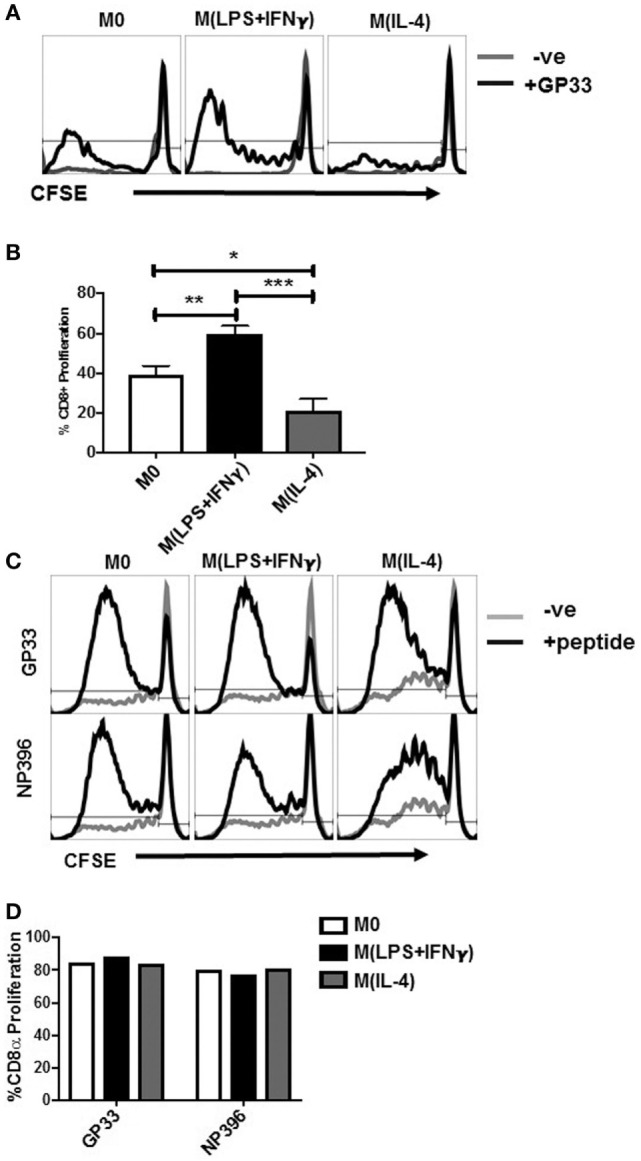
M(IL-4) Sp-MΦ poorly support epitope-specific CD8^+^ T-cell proliferation. Polarized Sp-MΦ were pulsed with the LCMV GP33-41 peptide and cultured with CFSE labeled splenocytes from LCMV immune mice (10:1 ratio) for 4 days in the presence of 5 U/mL IL-2. **(A)** Representative histograms showing CFSE dilution in CD8^+^ T cells. **(B)** Graphical representation of % CFSE low, CD8^+^ T cells of three replicates from one representative experiment. *P < 0.05, **P < 0.0005, and ***P < 0.0005. **(C)** When the CD8^+^ T-cell proliferation experiments were carried out in culture supernatant containing 20 U/mL IL-2, no differences were observed in the ability of M(IL-4) Sp-MΦ to induce CD8^+^ T-cell proliferation when compared with either M0 or M(LPS + IFN-γ) cells. **(D)** Graphical representation of % CFSE low, CD8^+^ T cells from one of four independent experiments. CFSE, carboxyfluorescein succinimidyl ester; LCMV, lymphocytic choriomeningitis virus infection.

Upon successful activation, naïve CD8^+^ T cells undergo an estimated 10^4^- to 10^5^-fold expansion at their peak proliferation during activation (43). Other groups have demonstrated that M(IL-4) MΦ inhibit antigen-specific T-cell proliferation (28, 29). These publications utilized culture systems with M(IL-4) BM-MΦ and naïve P14 CD8^+^ T cells (specific for GP33-41) or anti-TCR and anti-CD28 antibodies to artificially stimulate naïve CD8^+^ T-cell proliferation in an antigen-independent fashion proliferation (28, 29). However, how M(IL-4) cells direct memory recall responses is unknown. Therefore, we asked how CD8 T cells from LCMV memory, non-transgenic mice would respond to antigen presentation by polarized Sp-MΦ. To assess this, we cultured peptide-pulsed either polarized or unstimulated Sp-MΦ with carboxyfluorescein succinimidyl ester (CFSE) labeled splenocytes from LCMV immune WT mice in the absence or presence of IL-2 (Figures 5A,B). Without exogenous IL-2 added to cultures, all the three MΦ populations failed to induce recall proliferation *in vitro* (data not shown), confirming previously published data by other groups (44). We then increased IL-2 culture concentrations to determine the minimum threshold of exogenous IL-2 required for T-cell expansion. At 5 U/mL, M0 and M(LPS + IFN-γ) MΦ induced 40 and 60% CD8^+^ T-cell proliferation, respectively (Figures 5A,B). In contrast, M(IL-4) induced the lowest proliferation (20%) by antigen-specific CD8^+^ T cells (Figures 5A,B).

We were able to restore the deficit in the stimulation ability of M(IL-4) cells by increasing the IL-2 levels in the culture system to 20 U/mL. This finding was not restricted to a single specificity of epitope-specific T cells, as both GP33-41- and NP396-404-specific T cells proliferated to similar levels when comparing M(IL-4) with either M(LPS + IFN-γ) or M0 (Figures 5C,D). Collectively, the data from Figures 2–5 demonstrate that although M(IL-4) cells are proficient at presenting antigens to CD8^+^ T cell and stimulate them to elicit IFN-γ release either as effector, memory cells, M(IL-4)-MΦ were poor inducers of their if IL-2 is not present at sufficient quantities in the environment.

### CD8^+^ T Cells Expanded for 6 Days by M(IL-4) Stimulators Exhibit Attenuated IFN-γ Secretion Compared with M0 or M(LPS + IFN-γ) Cells

From our observations above, we noted that although CD8^+^ T-cell proliferation was impaired compared with M0 and M(IL-4), approximately 20–30% of CD8^+^ T cell were still proliferating after peptide antigen presentation with M(IL-4) MΦ (Figure 5B). As CD8^+^ T cells proliferate, they progressively acquire CTL functions (45). To test whether the epitope-specific CD8^+^ T cells were fully functional after the expansion period, we restimulated the expanded T cells using a common (BMA) MΦ cell line as antigen presenting cells so that the only variable factor in the assay would be the expansion difference of the Sp-MΦ (23, 42, 46).

**Figure 6:**
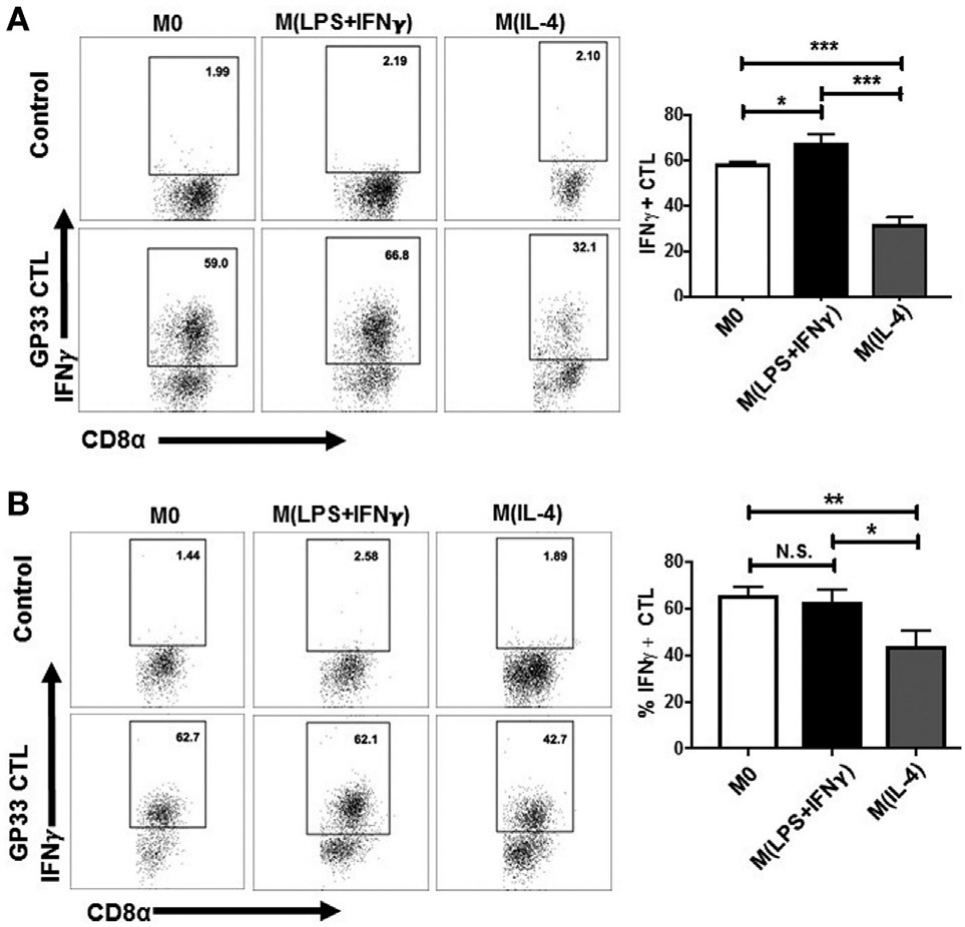
CD8^+^ T cells expanded by M(IL-4) stimulators exhibiting attenuated IFN-γ secretion. Sp-MΦ [M0, M(LPS + IFN-γ) or M(IL-4) cells] were pulsed with LCMV GP33-41 and cultured with CFSE-labeled splenocytes from LCMV immune mice (10:1 ratio) for 6 days in the presence of 5 U/mL IL-2 **(A)** or 10 U/mL IL-2 **(B)**. Expanded cells were restimulated with GP33-41 pulsed BMA or unpulsed controls for detection of IFN-γ production by ICS in a restimulation assay to detect their epitope-specific activation. Graphs show mean ± SD from three replicates where *P < 0.05 and ***P < 0.0005. CFSE, carboxyfluorescein succinimidyl ester; LCMV, lymphocytic choriomeningitis virus infection.

Consequently, we cocultured the cells *in vitro* for 6 days using 5 U/mL IL-2 because this was the concentration at which we observed minimal M(IL-4)-induced CTL expansion (Figure 5A). We then tested for IFN-γ production by ICS as described earlier, but this time using the BMA cell line as APC to present the LCMV peptide to T cells (Figure 6A).

M0 and M(LPS + IFN-γ)-expanded CTL were ~60% IFN-γ positive, whereas M(IL-4)-expanded CTL displayed lower level of ~30%. This indicates that in addition to M(IL-4) cells being poor expanders of CD8^+^ T cells, the CTLs that were able to expand were not potent effector cells. Upon increasing culture conditions to 10 U/mL of IL-2 (Figure 6B), we noted a partial restoration (an increase of approximately 10%) of IFN-γ production by CD8^+^ T cells cocultured with the M(IL-4) stimulators. This finding suggests that exogenous IL-2 can overcome stimulatory deficits in M(IL-4) cells, implying that M(IL-4) cells can dampen the proliferation and subsequent cytokine synthesis in CD8^+^ T cells through antigen presentation when IL-2 levels are low or limiting in the environment.

### Activated CD4^+^ T Cells from LCMV Immune Mice are Not Sufficient to Enhance the M(IL-4) Expansion of CD8^+^ T-Cell Proliferation

CD4^+^ T helper cells play an important role in shaping efficient CTL, CD8^+^ T-cell memory, and recall responses (47). Evidence suggests that for efficient CD8^+^ T-cell responses to occur, the APC must be helped by CD4^+^ T cells (48). In particular, direct engagement between CD40 and CD40L activates APC to enable IL-2 production for memory CD8^+^ T-cell proliferation (49). Given that IL-2 rescued CD8^+^ T-cell proliferation in M(IL-4) MΦ cultures in our experimental model, we reasoned that activated CD4^+^ T from an *in vivo* LCMV infection would compensate for the M(IL-4) ability to support CD8^+^ T cells in our model when the exogenous IL-2 levels are limiting in culture.

**Figure 7:**
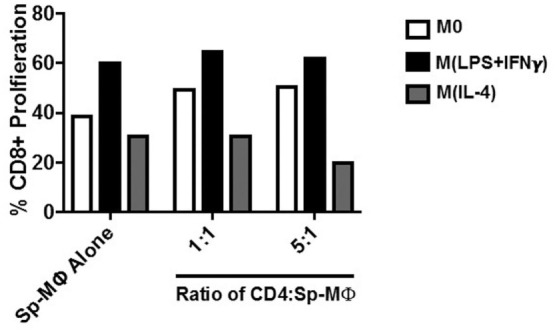
Activated CD4^+^ T cells from LCMV immune mice fail to enhance the M(IL-4) expansion of CD8^+^ T-cell proliferation to levels obtained with the M(LPS + IFN-γ) cells. CD4^+^ T cells were negatively sorted from LCMV-infected mice (20,000 pfu) on day 4 post-infection as described in Section “Materials and Methods.” The CD4^+^ T cells were added in different ratios to a polarized, GP33-41 pulsed Sp-MΦ mixed with splenocytes in culture containing 5 U/mL IL-2. As in Figure 5B, after 4 days of coculturing, the CD8^+^ T cells were tested for CFSE dilution as described before. CFSE, carboxyfluorescein succinimidyl ester; LCMV, lymphocytic choriomeningitis virus infection.

To test this hypothesis, we sorted CD4^+^ T cells from LCMV-infected mice on day 4 because it has been shown that CD69 expression levels are near their peak after virus infection *in vivo* (50). We cocultured the sorted CD4^+^ T cells with the MΦ plus CD8^+^ T cells during the CFSE proliferation assays in our model, when exogenous IL-2 levels are limiting. Although we observed that addition of CD4^+^ T cells enhanced the ability of M0 cells to increase CD8^+^ T-cell proliferation (from 38 to 50%), there was no positive influence in the M(IL-4) cultures (Figure 7) possibly because they may require additional help. Interestingly, no further proliferation (60%) was noted with M(LPS + IFN-γ) cells when we included the helper cells, indicating that a possible maximum proliferation had already been reached at such IL-2 levels without the CD4^+^ T cells present in the culture.

## Discussion

It is now understood that polarized MΦ can modulate the outcome of infection and play a role in exacerbating or combating multiple diseases (4, 10). We previously reported that M-CSF induces *in vitro* differentiation of Sp-MΦ into cells that resemble splenic red pulp MΦ (23, 51). Further characterization revealed that Sp-MΦ are effective antigen presenting cells capable of direct and cross presentation (23). Additionally, mature Sp-MΦ remain plastic and can be induced to M(LPS + IFN-γ) and M(IL-4) phenotypes, similar to BM-MΦ (8).

Several groups have reported on the ability of M(IL-4) polarized cells to negatively regulate CD4^+^ T cells (6, 9, 28, 52–54). In addition, M(IL-4)-polarized BM cells were shown to inhibit CD3/CD28-activated naïve CD8^+^ T-cell proliferation, and impair proliferation of naïve LCMV-specific transgenic (P14) CD8^+^ T cells in a helminth/norovirus coinfection model (28, 29). However, not much is known regarding how polarized MΦ influence antigen-specific CD8^+^ T-cell IFN-γ secretion and memory CD8^+^ T-cell recall responses after antigen presentation has ensued, particularly during viral infection. To this end, using the well-defined LCMV model, our study uncovers a dichotomous effect of M(IL-4) MΦ on CD8^+^ T-cell proliferation and effector molecule release.

We demonstrate that polarized M(IL-4) MΦ present MHC-I-restricted peptides to activate CD8^+^ T cells and stimulate IFN-γ expression. LCMV infected all three subsets of MΦ as detected by LCMV-NP expression 24 h post-infection. However, we observed a substantial reduction of LCMV-NP in M(LPS + IFN-γ) cells. This is likely owing to the upregulation IFN-γ-induced anti-viral genes inhibiting the replication of capacity of LCMV described (55). M(IL-4) cells effectively processed and presented *de novo* synthesized antigens to activate GP33-41- and NP396-404-specific CTL. However, when polarized cells were cultured with splenocytes cells from LCMV-immune mice, M(IL-4) cells stimulated the lowest level of CD8^+^ T-cell proliferation, a deficit that was overridden by increasing the levels of exogenous IL-2 added to the culture system. Collectively, these observations highlight how polarized MΦ modify CD8^+^ T-cell function and are of particular importance to the design of cell-based immunotherapies.

The suppressive effects of M(IL-4) MΦ on T-cell proliferation have been reported in the literature where M(IL-4) cells prevented proliferation of EL4 and D10.G4 T cells through an undefined cell-contact-dependent mechanism during nematode infection (52). Subsequent gene analysis revealed several M(IL-4)-specific markers including program death ligand (PD-L2), resistin like molecule (RELM)-α and YM-1, regulating CD4^+^ T-cell and CD8^+^ T-cell proliferation (28, 29, 54). Moreover, *in vitro* M(IL-4) MΦ express high levels of Arginase-1 and can also interrupt TCR signaling through l-arginine deprivation (56). For instance, coculture of M(IL-4) MΦ with Jurkat T cells or spleen T cells for 24 h resulted in the downregulation of CD3ζ T-cell surface expression and inhibition of CD4^+^ T-cell proliferation (56–58). Moreover, l-arginine deprivation yields a G_0_–G_1_ arrest by preventing increases of cyclin D3 and cyclin-dependent kinase 4 (CDK4) levels (58). Thus, M(IL-4) cells have the ability to modulate T-cell proliferation by contact-dependent and contact-independent mechanisms.

In our system, we observed a significant reduction of antigen-specific memory CD8^+^ T-cell proliferation when stimulated with M(IL-4) cells compared with M0 or M(LPS + IFN-γ) when employing IL-2 at limiting concentrations. Interestingly, supplementing the coculture medium with additional IL-2 restored CD8^+^ T-cell proliferation and subsequent IFN-γ secretion upon restimulation. The most likely explanation for this finding is likely due to the ability of IL-2 to tune TCR sensitivity and regulate cell-cycle progression. With regard to TCR sensitivity, exogenous IL-2 restores basally depressed CD3ζ expression in patients with chronic myeloid leukemia (59). Moreover, it was recently demonstrated that IL-2 reduces the threshold of activation in CD8^+^ T cells promoting responsiveness to low antigen levels (60). Therefore, by adding IL-2 we ostensibly improved the TCR sensitivity to antigen. In terms of cell cycle, IL-2 enhances expression of cyclin D3 and CDK4, and activates CDK2 promoting cell-cycle progression into S phase (61, 62). Therefore, it is plausible that additional IL-2 overrides the M(IL-4) cell-induced CD8^+^ T-cell impairments by increasing CD3ζ expression and promoting entrance into cell cycle. In agreement with this notion, delivery of IL-2 complexed to anti-IL-2 monoclonal antibody breaks established CD8^+^ T-cell tolerance in an FBL model of murine leukemia (63). As such, future research into the biological mechanisms of our reported novel finding that IL-2 can overcome M(IL-4) stimulatory deficits CD8^+^ T cell is of immense interest to the immunotherapy field.

Another novel finding that is reported here is the ability of the polarized MΦ to elicit IFN-γ production by antigen-specific CTL following peptide stimulation or LCMV infection. Given the impairment of CD8^+^ T-cell TCR signaling described above following M(IL-4) coculture, we anticipated M(IL-4) cells to poorly induce IFN-γ secretion from CTL (57, 59). A limitation of these studies was that they did not address the consequence of short-term (<24 h) M(IL-4) MΦ–T-cell interactions on CD3ζ expression (57, 59). However, the duration of our cytokine detection assays (approximately 6 h) allows us to separate the short-term and long-term impacts of M(IL-4) cells on CD8^+^ T-cell effector function and memory CD8^+^ T-cell proliferative response.

In our system, the observed ability of M(IL-4) cells to stimulate CTL IFN-γ release is likely attributed to the lower threshold of CTL activation for cytokine production compared with proliferation (64). It was previously demonstrated that low concentrations of TCR ligands result in IFN-γ, but not IL-2 production nor proliferation in CD4^+^ T cells (64). However, as ligand concentration increases, so does the diversity of cytokine response and proliferation levels (64, 65). This implies that additional characteristic of polyfunctional CD8^+^ T cells requires a successive increase in signaling threshold. Conceptually, this might serve as a safeguard to control against unwanted CD8^+^ T-cell effector activity and cytokine production.

In summary, our results uncover a previously unknown ability of M(IL-4) cells to induce IFN-γ secretion by CTL and yet negatively regulate memory CD8^+^ T-cell proliferation compared with other forms of MΦ employed in this study. Thus, it is plausible that M(IL-4) cells possess novel undefined characteristics that need to be uncovered regarding how they may regulate immunity during infections.

## Materials and Methods

### Animals, Cell Lines, and Virus

C57BL/6 (H-2^b^) mice (6–8 weeks) were purchased from (Jackson Laboratories) and were kept under specific pathogen-free conditions. Animal experiments were carried out in accordance with the guidelines of the Canadian Council of Animal Use and with approval from Queen’s University Animal Care Committee. The BMA cell line BMA3.1A7 (a gift from Dr. K. Rock, University of Massachusetts Medical School, Worcester, MA, USA) is an adherent murine MΦ cell line generated from the BM of female C57BL/6 mice by overexpressing *myc* and *raf* oncogenes and has recently been characterized by our group to be a good model to study MΦ polarization (66, 67). For viral infections, mice were injected at indicated plaque forming units (PFUs) or LCMV-WE intraperitoneal in 200 µL of sterile PBS.

### Macrophage Generation and Activation

Bone marrow-MΦ and Sp-MΦ were generated as previously described (8, 23, 51). Briefly, cells (for both BM-MΦ and Sp-MΦ) were cultured in 6 well plates (Corning) with conditioned RPMI 1640 (Gibco) medium (CM) supplemented with 10% fetal calf serum (Fisher Scientific), M-CSF media and 50 µg/mL gentamycin. On days 3 and 5 of culturing, non-adherent cells were removed and fresh CM was added. Sp-MΦ and BM-MΦ were cultured for 6–7 days in total before activation and testing for their functions as described below. For M(LPS + IFN-γ) MΦ: cells were primed with IFN-γ (25 ng/mL for 16–18 h; Shenandoah Biotechnology) followed by *Escherichia coli* LPS (O55:B5, 100 ng/mL for 6 h; Sigma-Aldrich). To induce M(IL-4) MΦ, polarization cells were treated with rIL-4 (20 ng/mL for 18–24 h; Shenandoah Biotechnology). Unstimulated control MΦ (M0) were placed in RPMI 10% FCS for 18–24 h.

### Assessment of Arginase and Inducible Nitric Oxide Activity

M(LPS + IFN-γ) polarization was determined by assessing NO production using Griess reagent as published previously (8, 24). MΦ (200,000 cells/well) were seeded in a round-bottom 96-well plate and activated in phenol-red free RPMI, where nitrite concentration was determined by measuring OD at 540 nm using the Varioskan microplate reader. Sodium nitrite was used for the standard graph (0–100 µM) to calculate nitrite concentrations in the test samples and was purchased from Fisher Scientific (Whitby, ON, Canada).

M(IL-4) polarization was assessed by arginase activity as described (8, 68). Activated MΦ were resuspended in lysis buffer (0.1% Triton-X, 25-mM Tris–Cl, pH 8.0) purchased from Sigma (Oakville, ON, Canada) and incubated for 30 min at 4°C followed by centrifugation at 10,000× *g* for 20 min at 4°C. Protein content in supernatant was determined using Bradford reagent (Bioshop) and sample concentrations were equalized to 100 µg/mL. For the reaction, 100 µL of sample was added to 1.5-mL eppendorfs followed by 10 μL of 10-mM MnCl_2_ an incubation at a 55°C for 10 min. 100 µL 0.5-M l-arginine solution (pH 9.7) was then added before incubation for 1 h at 37°C. To stop the reaction, 800 µL of acid solution (7:3:1; H_2_O:H_2_PO_4_, 85%: H_2_SO_4_, 95%) and 40 µL of α-isonitrosopropiophenone (ISPF) (9% w/v in absolute ethanol) were added. Samples were heated at 100°C for 30 min and urea concentration was determined with the help of a urea standard graph (0–25 M) by measuring OD at 550 nm using a Varioskan spectrophotometric microplate reader.

### Cytokine ELISA Assay

Uninfected or LCMV-infected (MOI 3: 24 h) were seeded at 2–3 × 10^6^ cells/mL into 6-well plates and incubated at 37°C for 24 h. After incubation, ELISAs were performed on collected supernatants. The levels of IL-6, IL-10, IL-12p40, and TNF-α were measured in accordance with R&D systems manufacturer’s instructions.

### Reverse-Transcriptase Polymerase Chain Reaction (RT-PCR)

Total RNA was extracted from primary, LCMV-infected cells using TRI reagent (Molecular Research Center Inc.). RT reaction was carried out using RT master mix with reagents obtained from Froggabio (North York, ON, Canada). PCR was performed using Taq 5X Master Mix (Froggabio) and the following primers (Forward and Reverse) obtained from Integrated DNA Technologies (Coraville, IA, USA) for LCMV Nucleoprotein (F: 5′-TCC ATG AGA GCA CAG TGC GGG GTG AT-3′, R: 5′-GCA TGG GAG AAC ACG ACA ATT GAC C-3′) and 18S control (F:5′-AAACGGCTACCACATCCAAG-3′, R: 5′-CCTCCAATGGATCCTCGTTA-3′).

### Flow Cytometry

Cells were stained with a combination of surface marker antibodies detailed below. Primary direct staining was performed with antibodies purchased from Biolegend: FITC anti-CD86, clone RMMP-2; FITC anti-MHC-II (I-A/I-E), clone M5/114.15.2; FITC anti-CD25, clone 3C7; PE anti-MHC-I, clone AF6-88.5; PE Biotin anti-CD80, clone 1610A1; PE anti-PDL1, clone 10F.9G2; PE anti-CD137, clone 17B5; APC/Cy7 anti-CD69, clone H1.2F3. For indirect staining antibodies were purchased from Biolegend: Biotin anti-IFN-γ, clone XMG1.2; from eBioscience: Biotin anti-SIINFEKL/H2-K^b^, clone 25-D1.16. Here, applicable cells were stained with secondary Streptavidin-FITC (Invitrogen) or FITC anti-rat IgG, clone Poly4054 (Biolegend). Staining was carried out for 20–30 min at 4°C in FACS buffer containing 0.5% sodium azide in PBS. Samples were acquired using the Epics XL-MCL flow cytometer (Beckman Coulter, Miami, FL, USA) and analyzed using FlowJo software. Fold change in MFI was calculated by using the following formula: fold change = [(SIINFEKL MFI − Negative MFI)/Negative MFI], where “SIINFEKL” refers to cells that were pulsed with SIINFEKL peptide and “Negative” refers to unpulsed cells.

### Induction of LCMV-Specific CD8^+^ T Cells

Peptide-specific short-term T-cell lines were generated as previously described (23, 24, 42, 46). Splenocytes isolated from LCMV immune mice (30 days post-infection) were subjected to ficoll-gradient lymphocyte enrichment. Enriched T cells were cocultured with γ-irradiated peptide-loaded (GP33-41, NP396-404; 10^−7^M) BMA at a ratio of 10:1 in RPMI (10% FCS, 50-µM β-mercaptoethanol, 20 U/mL rIL-2, 50-µg/mL gentamycin). After 5 days, medium was isolated and ficolled to remove dead APC and enriched cells were re-seeded in a new 6-well plate with fresh medium for 2–3 days before use.

### Antigen Presentation Assays

In order to compare direct antigen presentation by activated MΦ, we utilized LCMV-specific T cells, either *in vitro* generated LCMV CTL or the B3Z hybridoma T cells. For *ex vivo* stimulation of LCMV-specific CD8^+^ T cells by polarized MΦ, splenocytes isolated from an LCMV-infected mouse on day 8 and were restimulated with GP33-41 or NP396-404 (10^−7^ M) peptide-pulsed MΦ (ratio of 10 splenocytes: 1 APC) for 2 h. ICS for IFN-γ production was then performed as described below. LCMV-specific CTL were cultured with activated MΦ (1:1) that were pulsed with decreased peptide molarity (GP33-41/NP396-404: 10^−7^ to 10^−9^ M) or infected with LCMV-WE (MOI 3 or 5) for various time points for 4 h in the presence of Brefeldin A (10 µg/mL; Sigma-Aldrich).

### B3Z Assay

B3Z CD8^+^ T-cell hybridoma cell line specific for OVA residues 257–264 (SIINFEKL) presented on murine MHC-I (H2-K^b^) was also used (36, 69). Cells were cultured in IMDM medium containing 500 µg/mL G418 to ensure positive selection of reporter cells until time of experiment. For antigen presentation assays, APC were pulsed with SIINFEKL peptide (10^−7^ or 10^−9^M) at 37°C for 2 h before extensive washing in warm PBS. Thereafter, the APC were cocultured with B3Z in a 96-well round-bottom plate (Thermo Scientific) at a ratio of 1:1 for 18 h in IMDM medium (5% FCS) at 37°C. For Laz-Z detection, Z Buffer (150 µL), containing 0.125% NP-40, 9-mM MgCl_2_, 100 mM of β-mercaptoethanol, and 5-mM ONPG, was added to the B3Z:APC cell pellets and incubated at 37°C for 4 h to allow for colorimetric change. Thereafter, 100 µL of buffer was transferred to a flat-bottom 96-well plate for absorbance measurement at 410 nm using a Varioskan plate reader.

### Detection of LCMV-NP

For LCMV-NP detection, infected cells were fixed and permeabilized with PBS containing 4% paraformaldehyde and 0.5% saponin for 20 min at room temperature. Cells were washed in PBS with 0.25% saponin and incubated for 1 h with rat anti-LCMV-NP Ab (clone VL4) supernatants (a gift from Dr. M. Groettrup, University of Constance, Germany) to detect NP expression (70). After washing twice, FITC-conjugated goat anti-rat IgG Ab (Invitrogen) was incubated with the cells overnight at 4°C. In separate experiments, propidium idodide (1 µg/mL; Sigma Aldrich, Oakville, ON, Canada) was added to uninfected and LCMV-infected samples for assessment of cell death. Data were acquired with the Epics XL MCL (Beckman) and analyzed with the FlowJo software.

### *In Vitro* CD8^+^ Proliferation Assay

Splenocytes were harvested from LCMV-WE immune (30 days post-infection) and lymphocytes were purified by ficoll-gradient centrifugation with lymphocytes separation medium (Fisher, Whitby, ON, Canada). Purified cells were labeled with CFSE (0.4 µM) for 15 min at 37°C then washed twice in warm PBS. Lymphocytes were cultured in a 96-well flat-bottomed plate with GP33-41 or NP396-41 peptide pulsed polarized MΦ for 3–6 days (at decreasing concentrations of recombinant IL-2) before staining for CD3 and CD8α expression and assessed for CFSE staining by FCM. Percent proliferation was determined by calculating the % of CD8^+^ CFSE^Low^ cells compared with unstimulated CFSE labeled controls (CFSE^Hi^). For the CD4^+^ T-cell coculture experiment: CD4^+^ T cells were negatively selected for by gating out B220^+^, CD11b^+^, F480^+^, and CD8^+^ cells from the spleen using a BD FACS Aria III sorter (Beckman-Dickinson). CD4^+^ T cells were added to MΦ^+^ CFSE labeled splenocyte at the indicated ratio and incubated for 4 days.

### Statistical Analysis

Statistical analysis was performed using Prism 7.0 (La Jolla, CA, USA) with unpaired (Student’s) *t*-test.

## Ethics Statement

Mice were used according to Canadian Council of Animal Care guidelines to isolate primary cells and the protocol for this work was approved by the Office of University Animal Care Committee at Queen’s University (Protocol no. 1536).

## Author Contributions

SB and RM contributed to the design of the project and writing of the paper. RM, AB and KS performed experiments.

## Conflict of Interest Statement

The authors declare that the research was conducted in the absence of any commercial or financial relationships that could be construed as a potential conflict of interest.
