# Corrigendum: M(IL-4) tissue macrophages support efficient interferon-gamma production in antigen-specific CD8+ T cells with reduced proliferative capacity

**DOI:** 10.3389/fimmu.2023.1233307

**Published:** 2023-06-28

**Authors:** Rylend Mulder, Andra Banete, Kyle Seaver, Sameh Basta

**Affiliations:** Department of Biomedical and Molecular Sciences, Queen’s University, Kingston, ON, Canada

**Keywords:** polarized macrophages, major histocompatibility complex, interleukin-4, interferon-gamma, T cells, lymphocytic choriomeningitis virus infection

In the published article, there was an error in Name of Figure/Table as published. During the final version of figure submission, one plot labelled (TNF) was mistakenly duplicated in **Figure 1C**. The corrected Name of Figure/Table and its caption appear below. The authors apologize for this error and state that this does not change the scientific conclusions of the article in any way. The original article has been updated.

**Figure 1:**
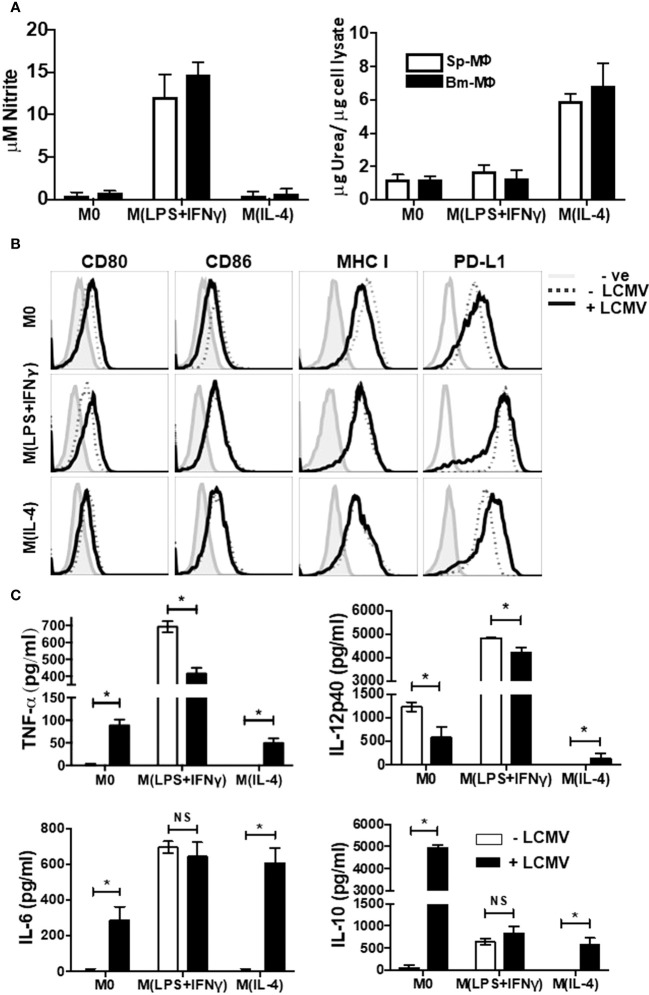
Immunophenotyping of Polarized Macrophages. Activated BM-MΦ or Sp-MΦ populations were polarized into M(LPS + IFN-γ) (25 ng/ml IFN-γ + 100 ng/ml LPS), or M(IL-4) (20 ng/ml IL4) or left un-stimulated. **(A)** Nitrite detection after BM-MΦ or Sp-MΦ were polarized into M(LPS + IFN-γ) or M(IL-4) or left un-stimulated (left panel). Supernatants were collected before testing them for nitrite production using the Greiss reaction. The OD was measured using Varioskan plate reader to quantify nitrite production after comparing the values to the standard curve. In the right panel, urea production was measured in polarized BMinfected with LCMV-WE (MOI 5M-MΦ and Sp-MΦ samples to monitor arginase activity indicative of M(IL-4) polarization. Values are represented as μg urea corrected to μg cell lysate. Data shown and error bars are the mean ± SD from one representative experiment out of three. **(B)** Staining profiles of activated polarized BM-MΦ and Sp-MΦ populations that were either controls or infected with LCMV-WE (MOI 5 for 24 h). Histograms show surface staining for CD80, CD86, MHC I or PD-L1 in the various MΦ populations compared to the isotype control (-ve). Data shown are representative from one of two experiments. **(C)** Cell supernatants from LCMV uninfected or LCMV infected (24 h) polarized Sp-MΦ were subjected to ELISA for quantification of TNF-α, IL-12p40, IL-6 and IL-10. Graphical data show mean ± SD from two independent experiments containing two experimental replicates.

