# Supplementary material for: M(IL-4) Tissue Macrophages Support Efficient Interferon-Gamma Production in Antigen-Specific CD8+ T Cells with Reduced Proliferative Capacity
Source: Front Immunol. 2017 Nov 30;8:1629. doi: 10.3389/fimmu.2017.01629 (PMC5714867; doi:10.3389/fimmu.2017.01629)
Supplement: Supplementary file 1 [file Data_Sheet_1.PDF]

Supplementary Material

M(IL-4) Tissue Macrophages support efficient Interferon-Gamma Production in Antigen-Specific CD8+ T cells with Reduced Proliferative Capacity.

Rylend Mulder, Andra Banete, Kyle Seaver, Sameh Basta*

*** Correspondence:** bastas@queensu.ca

Supplementary Figures

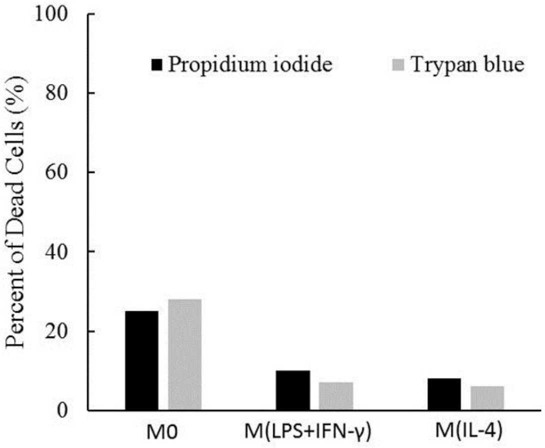

Supplementary Figure 1. The percentage of propidium dead cells due to viral infection in the three types of macrophages.

Tissue derived Sp-MΦ (M0, M(LPS+IFN-γ) and M(IL-4)) were left uninfected or infected with LCMV-WE (MOI 3) for 24 h before testing for cell viability by using the trypan blue dye (counted with microscopy) or propidium iodide (PI) staining (acquired with flow cytometry). The percentage of (PI +ve) cells before infection for each population (M0 = 14%, M(LPS+IFN-γ) = 27% and M(IL-4) = 18%) was subtracted from the percentage of PI positive cells after infection to compare cell death due to viral infection. In general, M(LPS+IFN-γ) and M(IL-4) cells showed similar increased cell death due to the viral infection with M0 showing more cell death. The data indicate that similarity in antigen presentation in the data obtained in 3B was not due abnormal death in M(LPS+IFN-γ) cell compared to M(IL-4) after viral infection. Representative data are depicted from one of two independent experiments.

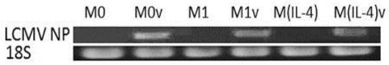

Supplemental Figure 2. Detection of viral genes early after infection in macrophages.

To check for relative levels of infection at early time points, the three MΦ populations were infected with LCMV (MOI 3) for 1.5 h before detection of LCMV-NP RNA by RT-PCR. PCR amplification products (25 cycles) of LCMV-NP and 18S control from uninfected (M) or LCMV-infected macrophages (Mv). We could not detect any signal for LCMV NP if the PCR amplification cycles were lowered to 20 cycles, indicating that what we detected was near the threshold of detection and not the result of over-amplification. Uninfected cells did not show any signal for LCMV-NP, while similarly evident bands were detected in all infected cells at this early time point post infection. This confirmed that viral entry and infection in the various cells were comparable and that none of the three cell populations were more permissible to viral entry that allowed for better antigen presentation later on. M0 = untreated cells, M(IL-4) = IL-4 treated, and M1 = M(LPS+IFN-γ).
